# PTSD as an Endothelial Disease: Insights From COVID-19

**DOI:** 10.3389/fncel.2021.770387

**Published:** 2021-10-29

**Authors:** Adonis Sfera, Carolina Osorio, Leah Rahman, Carlos Manuel Zapata-Martín del Campo, Jose Campo Maldonado, Nyla Jafri, Michael Allen Cummings, Steve Maurer, Zisis Kozlakidis

**Affiliations:** ^1^Department of Psychiatry, Loma Linda University, Loma Linda, CA, United States; ^2^Patton State Hospital, San Bernardino, CA, United States; ^3^Department of Psychiatry, Loma Linda University, Loma Linda, CA, United States; ^4^Instituto Nacional de Cardiologia Ignacio Chavez, Mexico City, Mexico; ^5^Department of Medicine, The University of Texas Rio Grande Valley, Edinburg, TX, United States; ^6^International Agency For Research On Cancer (IARC), Lyon, France

**Keywords:** PTSD, endothelia, SARS-CoV-2, COVID-19, mitochondria, lactate

## Abstract

SARS-CoV-2 virus, the etiologic agent of COVID-19, has affected almost every aspect of human life, precipitating stress-related pathology in vulnerable individuals. As the prevalence rate of posttraumatic stress disorder in pandemic survivors exceeds that of the general and special populations, the virus may predispose to this disorder by directly interfering with the stress-processing pathways. The SARS-CoV-2 interactome has identified several antigens that may disrupt the blood-brain-barrier by inducing premature senescence in many cell types, including the cerebral endothelial cells. This enables the stress molecules, including angiotensin II, endothelin-1 and plasminogen activator inhibitor 1, to aberrantly activate the amygdala, hippocampus, and medial prefrontal cortex, increasing the vulnerability to stress related disorders. This is supported by observing the beneficial effects of angiotensin receptor blockers and angiotensin converting enzyme inhibitors in both posttraumatic stress disorder and SARS-CoV-2 critical illness. In this narrative review, we take a closer look at the virus-host dialog and its impact on the renin-angiotensin system, mitochondrial fitness, and brain-derived neurotrophic factor. We discuss the role of furin cleaving site, the fibrinolytic system, and Sigma-1 receptor in the pathogenesis of psychological trauma. In other words, learning from the virus, clarify the molecular underpinnings of stress related disorders, and design better therapies for these conditions. In this context, we emphasize new potential treatments, including furin and bromodomains inhibitors.

## Graphical Abstract 1

Covid-19 triggers endothelial cell (EC) senescence and dysfunction, likely predisposing to PTSD by increasing microvascular permeability that enables the extravasation of stress molecules into the brain trauma-processing networks in amygdala, hippocampus and the medial prefrontal cortex. The virus upregulates host angiotensin II (ANG II) (via S1 antigen), usurps furin/plasmin (via S2 antigen), mitochondria (via ORF9b), and Sigma-1 receptors (Sig-1Rs) via NSP6. These structures, previously associated with PTSD, link the SARS-CoV-2 virus to increased susceptibility for stress related disorders. As ECs are major producers of brain derived neurotrophic factor (BDNF), a neurotrophin altered in PTSD, senescent ECs lower this molecule further, predisposing to stress related disorders.

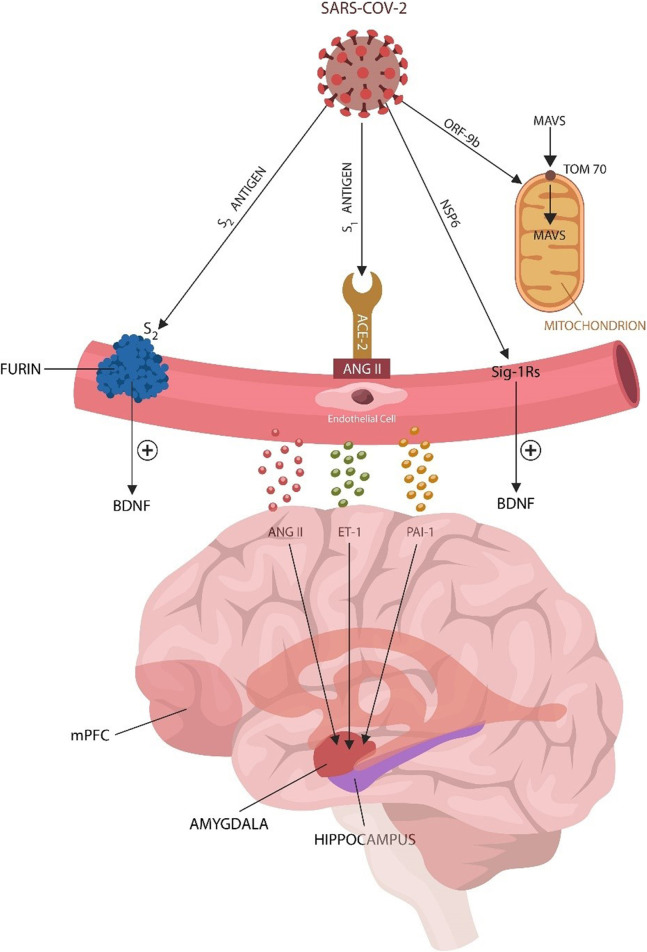

## Highlights

- The SARS-CoV-2 virus triggers endothelial senescence by usurping host serine proteases, the renin angiotensin system, and mitochondria.
- Senescent endothelial cells disrupt the BBB tight junctions, allowing stress molecules, including ANGc II, ET-1 and PAI-1, access to the amygdala, hippocampus, and mPFC, increasing the susceptibility for PTSD and other stress related disorders.
- Virus-damaged mitochondria predispose to PTSD by shifting cellular metabolism from OXPHOS to glycolysis in a Warburg effect.
- Virus-usurped furin and plasmin alter the pro-BDNF/BDNF ratio, predisposing to PTSD.

## Introduction

Severe acute respiratory syndrome coronavirus-2 (SARS-CoV-2), the etiological agent of COVID-19, spread rapidly throughout the world and was declared a pandemic in March 2020 (Wu and McGoogan, 2020). Restrictive measures, including mandatory isolation, social distancing, and absence of family support affected fragile populations, including the psychiatric patients, more than the society at large. In this group, many individuals have experienced recurrence of depression, anxiety, and use of substances, often culminating in posttraumatic stress disorder (PTSD) (Chamberlain et al., 2021; Janiri et al., 2021). Moreover, the experience of being hospitalized with COVID-19, facing intubation, tracheostomy, and the possibility of death, amplified the perception of life-threat, facilitating the development of stress related disorders (SRDs) as well as depression and anxiety (Dutheil et al., 2020; Tarsitani et al., 2021).

PTSD is a chronic debilitating syndrome that can originate in vulnerable individuals after exposure to real or threatened death, sexual assault, or severe illness. Impaired fear extinction and excessive anxiety are believed to drive the symptoms of this disorder (Myers and Davis, 2007). Clinical manifestations include re-experiencing the traumatic event(s), hypervigilance, exaggerated startle reflex, intrusive memories, nightmares, and dissociative phenomena (Lancaster et al., 2016).

Although primarily treated by psychiatrists, PTSD may be conceptualized as a systemic disease, considering its frequent association with medical conditions, including metabolic, autoimmune, and vascular disorders (Edmondson et al., 2013; Yapici-Eser et al., 2021). In this regard, the comorbidity of PTSD with cardiovascular and cerebrovascular disease, suggests that dysfunctional endothelia may play a key role in the pathophysiology of this disorder (Vaccarino et al., 2013; Chen et al., 2015; Grenon et al., 2016).

In the general population, about 8% of trauma-exposed individuals develop PTSD, while in military personnel and war veterans, the PTSD prevalence can reach up to 16% (Gates et al., 2012). Interestingly, some viral infections, including SARS-CoV-2, human immunodeficiency virus (HIV) and Ebola were associated with PTSD rates upward of 30%, indicating that these pathogens may interact directly with microvessels and/or the stress-processing centers of the brain (Lam et al., 2009; Siyahhan Julnes et al., 2016; de Solis et al., 2017; Spottswood et al., 2017; Kumar et al., 2021). Indeed, recent epidemiological data reported a PTSD prevalence of up to 30.2% in COVID-19 survivors, 39.5% in Ebola and 34% in HIV, suggesting that these viruses may directly target the cells of brain stress-processing pathways (Israelski et al., 2007; Bah et al., 2020; Janiri et al., 2021). In addition, as these pathogens contain a furin cleaving site (FCS), PTSD may be precipitated by the viral exploitation of furin and plasmin. These serine proteases were previously implicated in SRDs as they convert a precursor protein, pro-BDNF, into brain derived neurotrophic factor (BDNF) (Levin et al., 1997; Aksu et al., 2018; Miranda et al., 2019).

Numerous studies have associated PTSD with the stress mediators of the hypothalamic-pituitary-adrenal (HPA) axis and autonomic nervous system, the two main drivers of stress related phenotypes. However, here we only discuss the SARS-CoV-2-usurped renin-angiotensin system (RAS) that can also trigger this pathology (Chrousos and Zapanti, 2014; Griffin et al., 2014; Marinzalda Mde et al., 2014; Terock et al., 2019; Yu et al., 2019; Shkreli et al., 2020; Seligowski et al., 2021). Indeed, angiotensin receptor blockers (ARBs) and angiotensin converting enzyme inhibitors (ACEi) showed beneficial effects in both PTSD and severe COVID-19, suggesting that dysfunctional RAS may play an essential role in both conditions (Khoury et al., 2012; Marvar et al., 2014; Vian et al., 2017). For instance, the SARS-CoV-2 attachment to angiotensin converting enzyme 2 (ACE-2), disrupts the blood-brain barrier (BBB) by inducing senescence in cerebral endothelial cells ECs (Buzhdygan et al., 2020; Mehta et al., 2021; Rhea et al., 2021; Sanchez-Vazquez et al., 2021). This may enable ANG II to aberrantly activate its receptors in the amygdala, hippocampus, and the medial prefrontal cortex (mPFC), lowering the PTSD resilience (Marinzalda Mde et al., 2014; Wang L. et al., 2016; Terock et al., 2019; Yu et al., 2019; Shkreli et al., 2020). Increased BBB permeability facilitates endothelin-1 (ET-1) and plasminogen activator inhibitor 1 (PAI-1) extravasation and interaction with their respective receptors in the stress-processing pathways, predisposing to PTSD (Kurihara et al., 2000; Jiang et al., 2016; Chen et al., 2017a; Bouarab et al., 2021). Moreover, as mitochondria express ANG II type 1 receptors (AT-1Rs), the virus may disrupt these organelles, shifting metabolism, from oxidative phosphorylation (OXPHOS) to glycolysis and lactate accumulation (Barron et al., 2004; Doughan et al., 2008; Mellon et al., 2019; Sabbatinelli et al., 2019; Gordon et al., 2020; Sher et al., 2020; Icard et al., 2021). It has been established that lactate triggers flashbacks and panic attacks in PTSD patients, suggesting that unchecked glycolysis may account for the increased prevalence of SRDs in COVID-19 (Jensen et al., 1997; Johnson et al., 2013).

In summary, the SARS-CoV-2 viral antigens may upregulate ANG II, ET-1 and PAI-1, promoting mitochondrial dysfunction, EC senescence and increased BBB permeability. Extravasation of these stress-molecules into the brain parenchyma may reprogram the amygdala, hippocampus and mPFC, predisposing to PTSD.

In our previous work, we discussed ANG II-mediated mitochondrial dysfunction and premature EC senescence (Sfera et al., 2020, 2021a,b). Here, based on the SARS-CoV-2 interactome, we go a step further, linking PTSD vulnerability to virus-exploited furin, Sigma-1 receptors (Sig-1Rs), plasmin, bromodomains and mitochondria, moving from specific pathways to organelle structure. In other words, attempting to learn from the virus, may clarify the SRDs molecular underpinnings, and develop better therapies for patients with PTSD. We also discuss potential new treatments, including the inhibitors of furin and bromodomains.

## Stress, Aging Vessels, and the SARS-CoV-2 Virus

SARS-CoV-2 is an enveloped, positive-sense single-stranded RNA virus that ingresses human cells through several proteins, including ACE-2 (Bourgonje et al., 2020; Hoffmann et al., 2020). The virus consists a genome of about 29.9 kb, encoding for 29 structural, non-structural (NSP) and open-reading frame (ORF) antigens that interact with numerous human proteins, triggering pathological changes (Gordon et al., 2020). For example, virus-upregulated ANG II interacts with AT-1Rs on cerebral ECs, inducing premature senescence and increased BBB permeability (Fleegal-DeMotta et al., 2009; Marinzalda Mde et al., 2014; Marvar et al., 2014; Yu et al., 2019). A similar pathology is triggered by viral interaction with mitochondria, bromodomain 4 (BRD4), furin, plasmin, and Sig-1Rs (Table 1; Kunieda et al., 2006; Doughan et al., 2008; Gordon et al., 2020; Chamberlain et al., 2021). The connection between FCS and PTSD, as well as Sig-1Rs and PTSD are presented in detail below:

**TABLE 1:** Virus-host interactome: mechanisms of EC senescence.

Viral antigen	Human Protein	EC senescence mechanism	References
S2	Furin/plasmin	BDNF dysfunction	Gray and Ellis (2008); Gordon et al. (2020)
S**1**	ACE-2	Mitochondrial dysfunction	Gordon et al. (2020); Paris et al. (2020)
NSP6	Sigma-1	BDNF, Mitochondrial dysfunction	Hashimoto (2015); Gordon et al. (2020)
ORF9b	TOM70	Mitochondrial dysfunction	Gordon et al. (2020)
E	BRD2/BRD4	Mitochondrial dysfunction	Gordon et al. (2020); Johnson et al. (2020)

### Furin Cleaving Site and PTSD

Novel studies reported premature endothelial senescence in veterans and non-veterans with PTSD, indicating that BBB disruption likely plays a pivotal role in this disorder (Boscarino, 2008; Grenon et al., 2016; Thurston et al., 2018). Others have found that war veterans with PTSD, like older individuals, present with defective mitochondria, telomeres, furin/plasmin, and BDNF, connecting cerebral EC senescence with dysfunctional subcellular structures (Levin et al., 1997; Doughan et al., 2008; Gray and Ellis, 2008; Fleegal-DeMotta et al., 2009; Khoury et al., 2012; Grenon et al., 2016; Aksu et al., 2018; Connolly et al., 2018; Stein et al., 2018; Miranda et al., 2019; Seligowski et al., 2021; Cleveland et al., 2021).

**FIGURE 1:**
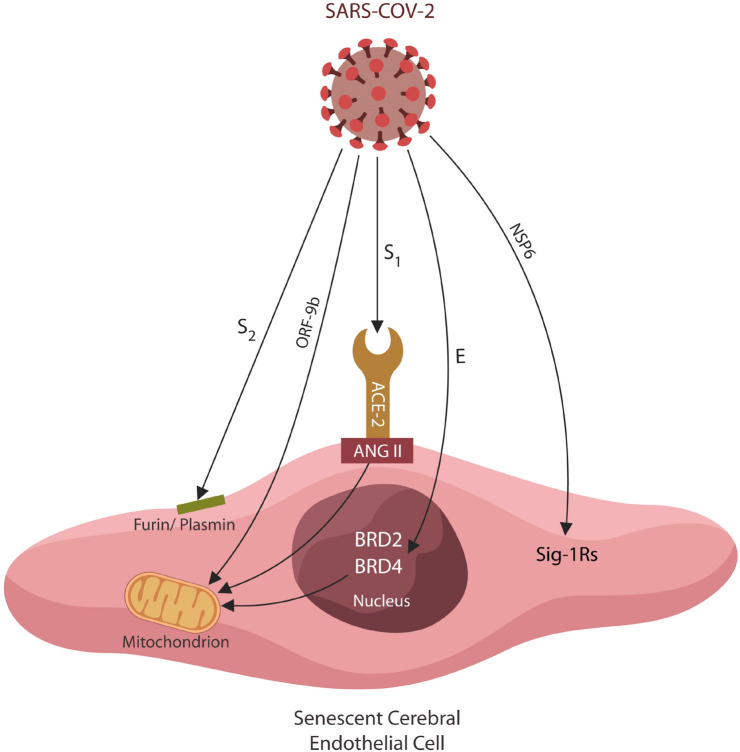
Several SARS-CoV-2 antigens interact directly with host EC proteins, inducing cellular senescence and altering the BBB permeability. Viral hijacking of furin/plasmin by antigen S2 impairs BDNF maturation, increasing PTSD susceptibility. The S1/ACE-2 attachment upregulates ANG II, inducing mitochondrial damage and cellular senescence. Viral ORF9b disrupts mitochondria directly, altering antiviral defenses and cellular metabolism. The SARS-CoV-2 antigen E exploits host epigenetic readers BRD-2 and BRD-4, altering the expression of mitochondrial proteins encoded in the nuclear DNA. Viral antigen NSP6 interacts with Sigma-1 receptors, inducing cellular senescence by an alternative pathway.

The SARS-CoV-2 antigen S1 usurps ACE-2, upregulating ANG II, while S2 protein exploits furin and plasmin (Table 1). Furin and plasmin are cell membrane serine proteases that under normal circumstances promote the maturation of BDNF, a neurotrophin involved in both PTSD and COVID-19 (Angelucci et al., 2014; Miller et al., 2017; Azoulay et al., 2020). Viral S2 antigen activates the FCS, hijacking host furin and depleting BDNF that in turn increases the risk of COVID-19 and SRDs (Gray and Ellis, 2008; Johnson et al., 2020; Table 1). Indeed, other viruses expressing FCS, including HIV and Ebola, were associated with a high PTSD prevalence, linking this motif to SRDs (Hallenberger et al., 1992; Volchkov et al., 1998; Figure 1). There are also PTSD comorbidities observed with further viral infections, however the scientific basis of those correlations remains an active field of research (Papić et al., 2011; Coughlin, 2012; Morais-de-Jesus et al., 2014). Moreover, as furin and plasmin regulate synaptic plasticity and the fibrinolytic system, an impaired tissue-type plasminogen activator (tPA)/PAI-1 ratio, may lower the resilience for both PTSD and COVID-19. Indeed, severe COVID-19 and impaired fear extinction were associated with tPA/PAI-1 dyshomeostasis (Bouarab et al., 2021; Zuo et al., 2021).

Another peripheral molecule that alters BDNF maturation is ET-1, a potent vasoconstrictor upregulated by ANG II (Lin Y.J. et al., 2014; Chen et al., 2017b; Ward et al., 2018; Notaras and van den Buuse, 2020; Diwakar et al., 2021). Since ET-1 was directly corelated with PTSD, its exploitation by the SARS-CoV-2 virus (via ANG II) may also contribute to the high comorbidity of PTSD and COVID-19 (Yammine et al., 2014; Fox et al., 2018). Interestingly, ET-1 is synthesized as a biologically inactive protein, proET-1, that requires processing by furin to convert to bigET-1 and subsequently to ET-1 (Denault et al., 1995). Along these lines, HIV, a FCS-containing virus, was associated with upregulated ET-1, likely accounting for the high PTSD comorbidity (Kanmogne et al., 2005; Neigh et al., 2016; Jain and Mehrotra, 2020).

Taken together, dysfunctional subcellular components, including furin/plasmin and BDNF, trigger cerebral EC senescence and BBB disruption. The FCS motif of the SARS-CoV-2 virus usurps human furin and plasmin, altering BDNF homeostasis that in turn predispose to SRDs (Kanmogne et al., 2005; Figure 1).

### Sigma-1receptors and PTSD

The SARS-CoV-2 antigen NSP6 exploits host Sig-1R, a positive BDNF regulator, likely disrupting synaptic plasticity and predisposing to SRDs (Fujimoto et al., 2012; Hashimoto, 2015, 2021; Xu et al., 2015; Gordon et al., 2020; Figure 1). In addition, as ECs are major sources of BDNF and senescent ECs are depleted of this neurotrophin, the susceptibility for both COVID-19 and PTSD is likely increased (Nakahashi et al., 2000; Descamps et al., 2018; Marie et al., 2018; Table 1).

Under physiological circumstances, Sig-1Rs agonists upregulate BDNF, protecting against EC damage triggered by either psychological stress and possibly the SARS-CoV-2 virus (Kimura et al., 2013). In addition, activated Sig-1Rs increase mitochondrial pregnenolone, protecting the organelle from psychological stress and virus-mediated dysfunction (Marriott et al., 2012) (see the section on mitochondria and neurosteroids). Indeed, the Sig-1Rs agonist, fluvoxamine, was shown to lower the intensity of stressful memories in SRD patients and to ameliorate COVID-19 outcomes (De Boer et al., 1992; Escalona et al., 2002; Pal et al., 2012; Hashimoto, 2013, 2021; Ji et al., 2017; Inserra, 2018; Sukhatme et al., 2021). Moreover, Sig-1R activating drugs were reported to protect against ANG II-induced EC damage, suggesting that combining ARBs/ACEi with Sig-1R agonists could have a superior therapeutic value in both COVID-19 and PTSD (Meunier and Hayashi, 2010; Hirano et al., 2014; Natsvlishvili et al., 2015; Liu et al., 2018; Lewis et al., 2019). Furthermore, virus-upregulated ANG II and hijacked Sig-1Rs may explain the early development of neurodegeneration observed in many PTSD patients (Yaffe et al., 2010; Mohlenhoff et al., 2017; Sobczak et al., 2021).

In response to stressors, cells undergo senescence, a state of proliferation arrest and active metabolism fueled primarily by glycolysis-derived lactate (Wiley and Campisi, 2016). Interestingly, under normal circumstances, ECs obtain over 80% of adenosine triphosphate (ATP) from lactate despite oxygen availability (Warburg effect), probably explaining the SARS-CoV-2 predilection for these cells (Sabbatinelli et al., 2019; Leung and Shi, 2021). Indeed, as senescent ECs upregulate lactate even more than their younger counterparts, they engender an optimal environment for viral replication (Schmid et al., 2007; Wong et al., 2017). Upon crossing into the brain parenchyma, lactate generates a local acidic microenvironment that may activate the acid-sensing ion channel-1a (ASIC1a), a protein implicated in SRDs (Coryell et al., 2009; Ziemann et al., 2009; Smoller et al., 2014; Quagliato et al., 2018). For example, low brain pH activates amiloride-sensitive cation channel, an member of the ASIC1a family, triggering PTSD symptoms (Taugher et al., 2017; Quagliato et al., 2018). Indeed, lactate was demonstrated to precipitate flashbacks and anxiety in PTSD and panic disorder patients, connecting unchecked glycolysis to the high prevalence of SRDs in COVID-19 survivors (Jensen et al., 1997; Filipović et al., 2020). Indeed, ASIC1a receptor antagonist, amiloride, was reported beneficial in anxiety and SRDs, further implicating glycolysis in these conditions (Pidoplichko et al., 2014; Battaglia et al., 2019). For this reason, amiloride nasal spray is considered therapeutic for PTSD as it readily crosses the BBB, inhibiting ASIC1a (Dwyer et al., 2009; Yellepeddi et al., 2020). Interestingly, under physiological circumstances, Sig-1R agonists also inhibit ASIC1a, indicating that drugs like fluvoxamine may counteract the detrimental effects of excessive brain lactate (Herrera et al., 2008). In consequence, a combination of fluvoxamine and amiloride may be a superior PTSD therapy compared to each drug individually.

Hijacking Sig-1Rs, the SARS-CoV-2 virus likely increases the detrimental effects of lactate on brain stress-processing pathways, explaining the high prevalence of SRDs in this infection (Vela, 2020; Figure 1). Moreover, ASIC1a receptors are abundantly expressed in cerebral arteries, suggesting that, like ANG II, upregulated lactate may increase the BBB permeability (Lin L.H. et al., 2014). This is in line with preclinical studies showing that lactate operates hand in hand with ANG II to engender anxiety (Shekhar et al., 2006; Yu et al., 2019).

Taken together, Sig-1R agonists, such as fluvoxamine, may reverse the negative effects of virus-upregulated ANG II on endothelia. As Sig-1R are downregulated by psychological and cellular stress, agonists at these receptors in combination with ARBs, ACEi or amiloride may offer superior therapeutic efficacy to patients with PTSD and severe SARS-CoV-2 (Spottswood et al., 2017; Hashimoto, 2021; Janiri et al., 2021).

## The SARS-CoV-2 Virus, Psychological Stress, BDNF and the Fibrinolytic System

Beyond the direct interaction of SARS-CoV-2 proteins with specific pathways in the interactome, the following paragraphs take a wider, more systematic perspective, focusing on psychological stress and BDNF as well as the fibrinolytic system.

### The Virus and BDNF

Brain derived neurotrophic factor is a growth factor belonging to the neurotrophin family that promotes angiogenesis, neurogenesis, and synaptic plasticity, enhancing the CNS recovery after insults (Alhusban et al., 2016). BDNF is synthesized as a biologically inactive precursor protein, pro-BDNF, that is activated upon furin or plasmin processing (Luchkina and Bolshakov, 2019).

Brain derived neurotrophic factor signals with tropomyosin receptor kinase B (TrkB), promoting hippocampal long-term potentiation (LTP), synaptic plasticity and memory formation (Jia et al., 2010; Leal et al., 2015). In contrast, pro-BDNF activates p75 neurotrophin receptor (p75NTR), inducing hippocampal long-term depression (LTD), dysfunctional synaptic plasticity and likely impaired fear learning (Rösch et al., 2005; Martinowich et al., 2012). Indeed, PTSD was associated with LTD, defective fear extinction, and impaired memory, likely accounting for the hypermnesia and dissociative amnesia documented in these patients (Graves et al., 2016; He et al., 2018).

In the adult brain, p75NTR is expressed almost exclusively in the cholinergic neurons of the basal forebrain and is altered by psychological stress, connecting neurotrophins to acetylcholine signaling (Woo et al., 2005; Martinowich et al., 2012; Green et al., 2013; Aksu et al., 2018; Boskovic et al., 2018; Ilchibaeva et al., 2018). Along these lines, neuroimaging studies in PTSD patients found abnormal activation of basal forebrain cholinergic neurons, suggesting that p75NTR may be upregulated (Hughes and Shin, 2011; Sherin and Nemeroff, 2011; Turchi et al., 2018). In addition, the adverse effects of acetylcholinesterase inhibitors often resemble PTSD symptoms, indicating that hyperactive cholinergic signaling may play a role in SRDs (Kaufer et al., 1998; McLay and Ho, 2007). Indeed, preclinical studies associated PTSD with dysfunctional p75NTR in basal forebrain (Martinowich et al., 2012). This is in line with the clinical studies in combat veterans with PTSD and non-veterans with anxiety and aggression that documented abnormal pro-BDNF/BDNF ratio, likely due to p75NTR upregulation (Matsuoka et al., 2015; Ilchibaeva et al., 2018; Kowiański et al., 2018).

Despite the established connection between BDNF and PTSD, the neurotrophin serum levels have been inconclusive, indicating that more studies are needed to clarify this issue (Angelucci et al., 2014; Martinotti et al., 2015; Mojtabavi et al., 2020). For example, low BDNF was found in individuals that developed PTSD shortly after stress exposure, while others found increased BDNF levels in patients with established PTSD (Angelucci et al., 2014; Mojtabavi et al., 2020). This discrepancy might be reconciled if BDNF is considered together with nitric oxide (NO) and peroxinitrate levels (Biojone et al., 2015; Banoujaafar et al., 2016). While NO itself lowers BDNF, peroxynitrate was shown to upregulate astrocytic growth factors, probably including the BDNF (Canossa et al., 2002; Vargas et al., 2004). Psychological stress was associated with the overexpression of nitric oxide synthase and peroxynitrate upregulation, linking oxidative stress with psychosocial trauma (Kumar and Chanana, 2017). On the other hand, NO has antioxidant properties and was reported to decrease anxiety and depressive-like behaviors in animal models (Kumar and Chanana, 2017). Interestingly, peroxynitrate is a TrkB receptor agonist that may compete with BDNF for the receptor site, accounting for the elevated BDNF levels documented in patients with chronic PTSD (Yuen et al., 2000; Amoureux et al., 2008; Krolow et al., 2014). Moreover, ebselen, a peroxynitrate scavenger, was found therapeutic in COVID-19, probably by restoring the physiologic BDNF/TrkB signaling, indicating a potential therapeutic value in PTSD (Chen et al., 2007; Amporndanai et al., 2021). Along these lines, several studies found that NO inhibitors, including methylene blue, can lower anxiety and depression, ameliorating many SRD symptoms (Chen et al., 2007; Zoellner et al., 2017). Furthermore, a polymorphism of NO gene, nitric oxide synthase 1 adaptor protein (NOS1AP) and a BDNF variant, Val66Met, were associated with severe PTSD, emphasizing the importance of NO/BDNF dialog in this disorder (Chen et al., 2006; Bruenig et al., 2017; Pitts et al., 2019). Indeed, a dysfunctional NO/BDNF interaction with resultant EC senescence, was found in both PTSD and COVID-19 critical illness (Azoulay et al., 2020; Nehme et al., 2020). Interestingly, NO is a furin inhibitor that may lower SARS-CoV-2 infectivity by denying this serine protease to the virus (Wolf and Morrison, 2017). For this reason, NO is currently in clinical trials for COVID-19 (NCT04388683).

Other furin inhibitors, including diminazene, are currently being evaluated for efficacy against COVID-19, suggesting that withholding furin from the SARS-CoV-2 virus may comprise a valuable therapeutic strategy (AbdelMassih et al., 2020; Wu et al., 2020). As diminazene also blocks ASIC1a channels, attenuating the detrimental effect of brain lactate, this agent may also be therapeutic in PTSD (Qaradakhi et al., 2020). Furthermore, spironolactone, another furin inhibitor, was found to protect against COVID-19 critical illness, suggesting a potential role in PTSD (Schmidt et al., 2017).

Aside from neurons, BDNF also protects ECs and type II pneumocytes (both cell types targeted by SARS-CoV-2), emphasizing that this neurotrophin likely attenuates viral replication (Takeda et al., 2013; Wilcox and Pitt, 2020). Indeed, COVID-19 critical illness was associated with low BDNF, while recovery was directly corelated with the levels of this growth factor (Azoulay et al., 2020). Moreover, as ACE-2 is essential for BDNF release, viral exploitation of this protein, likely impairs synaptic plasticity, predisposing to SRDs (Paris et al., 2020). On the other hand, ARBs and ACEi were shown to upregulate BDNF, improving synaptic plasticity, further linking dysfunctional RAS to PTSD (Ishrat et al., 2015; Alhusban et al., 2016; Andhavarapu et al., 2021; Table 2).

**TABLE 2:** Drugs with potential benefit in PTSD and mechanism of action.

Drug	Action mechanism	References
Amiloride	ASIC1a antagonist	Pidoplichko et al. (2014); Battaglia et al. (2019)
Fluvoxamine	Sig-1R agonist	Herrera et al. (2008)
ARBs/ACEi	Downregulate ANGII, upregulate BDNF and AQP-4, lower PAI-1 and ROS,	Fogari and Zoppi (2006); Matsushita et al. (2010); Dikalov and Nazarewicz (2013); Baluta and Vintila (2015); Ishrat et al. (2015); Chen et al. (2017c); Andhavarapu et al. (2021); Hashimoto (2021)
Ebselen	Peroxynitrate scavenger	Chen et al. (2007); Amporndanai et al. (2021)
NO	Furin inhibitor	Wolf and Morrison (2017)
Diminazene	Furin inhibitor, ASIC1a antagonist	Qaradakhi et al. (2020)
Spironolactone	Furin inhibitor	Schmidt et al. (2017)
Statins	PAI-1 inhibitors	Kellici et al. (2021)
BDNF (via MIND)	TrkB agonist	Padmakumar et al. (2021)
7,8-dihydroxyflavone	TrkB agonist	Sanz-García et al. (2016)
Lithium	TrkB agonist	Forster et al. (1995)
LM22A-4	TrkB agonist	Forster et al. (1995)
Fluoxetine	AQP-4 upregulation	Di Benedetto et al. (2016)
SS-31	Mitochondrial antioxidant	Tarantini et al. (2018)
Propranolol	Glycolysis inhibitor	Vaiva et al. (2003); Ganji and Reddy (2021)
Dichloroacetate (DCA)	Glycolysis inhibitor, mitochondrial protector	Kho et al. (2019)
Brexanolone	Mitochondrial and BBB protector	Pineles et al. (2018); Morrison et al. (2019)
Progesterone	NO upregulation?	Balan et al. (2019); Ney et al. (2019)
JQ1/SF2523	Senolytics, BRD inhibitors	Go et al. (2021)
Trauma-focused psychotherapy	T-cell enhancer	Morath et al. (2014)

### The Virus and Fibrinolytic System

**FIGURE 2:**
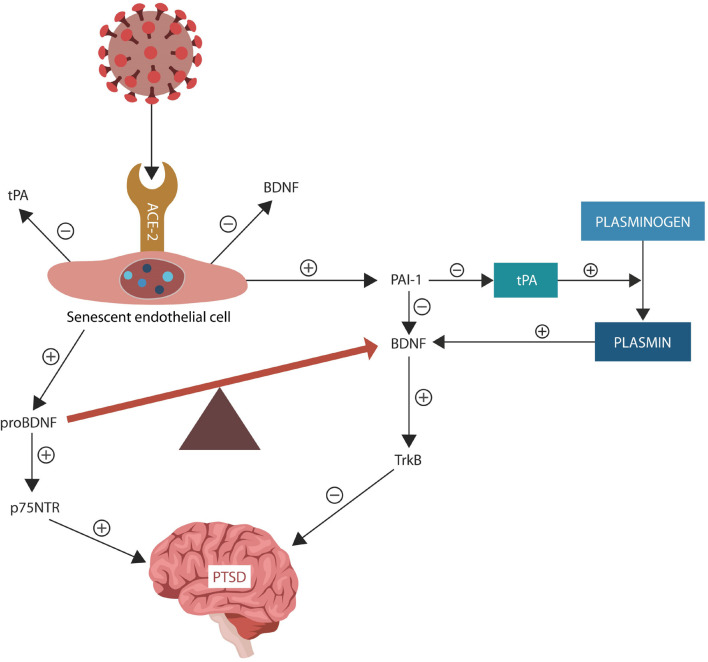
Under normal circumstances, tPA facilitates conversion of plasminogen to plasmin. PAI-1 is a tPA inhibitor that downregulates plasmin, a protein necessary for BDNF maturation. The SARS-CoV-2 interaction with ACE-2, increases ANG II inducing EC senescence. Senescent ECs upregulate PAI-1, lowering tPA and plasmin levels. SARS-CoV-2 antigen S2 usurps furin and plasmin (not shown), disrupting the conversion of pro-BDNF into BDNF, that in return impairs synaptic plasticity, predisposing to PTSD. Upregulated pro-BDNF increases PTSD vulnerability further.

The fibrinolytic system regulates thrombolysis (via plasmin activation) and neuroplasticity (via BDNF) (Wang X.L. et al., 2016). Both severe COVID-19 and PTSD were associated with upregulated PAI-1, a negative tPA regulator, indicating that targeting this protein may be therapeutic for both diseases (Cesari et al., 2010; Idell et al., 2017; Bouarab et al., 2021). Indeed, PAI-1 has been identified as a potential target in COVID-19 and its antagonists, such as statins, are currently in clinical trials (NCT04634799) (NCT04472611) (Bouarab et al., 2021; Kellici et al., 2021; Figure 2).

COVID-19-induced endothelial senescence and altered BBB may facilitate PAI-1 extravasation and interference with the stress-processing centers in amygdala, hippocampus, and mPFC. Several studies connected tPA/PAI-1 imbalance to PTSD, fear, anxiety, and depression, suggesting that restoring the fibrinolytic homeostasis may ameliorate the symptoms of SRDs (Pawlak et al., 2003; Bouarab et al., 2021). In addition, senescent ECs upregulate PAI-1, disrupting both plasmin activation and BDNF maturation, predisposing to PTSD (Pawlak et al., 2003; Tsai, 2017; Vaughan et al., 2017; Figure 2). On the other hand, ARBs and ACEi upregulate plasmin and downregulate PAI-1, promoting BDNF maturation and synaptic plasticity (Fogari and Zoppi, 2006; Baluta and Vintila, 2015; Chen et al., 2017c).

Attempts to increase BDNF levels with exogenous neurotrophin have been frustrating because of the unfavorable pharmacodynamic properties of this molecule, including poor BBB crossing. For this reason, a new modality, BDNF delivery via minimally invasive nasal depot (MIND), appears to be a promising PTSD treatment (Szekeres et al., 2010). In addition, several TrkB receptor agonists have been tested in veterans with PTSD, including the small molecule, 7,8-dihydroxyflavone, that showed beneficial effects in many patients (Padmakumar et al., 2021). In addition, as lithium displays TrkB agonism, it was found helpful, especially for the PTSD patients with impulsivity and affective instability (Sanz-García et al., 2016). Another TrkB agonist, LM22A-4, has been suggested for use in PTSD as it showed some positive results in animal models (Forster et al., 1995). Interestingly, candesartan was demonstrated to upregulate BDNF and promote angiogenesis, suggesting a potential treatment for dysfunctional endothelia and PTSD (Andero and Ressler, 2012; Table 2).

Taken together, psychological stress causes overexpression of nitric oxide synthase and peroxynitrate formation, disrupting the pro-BDNF/BDNF balance. Peroxynitrate scavengers and TrkB agonists, including intranasal BDNF, may have therapeutic value in SRDs.

## Of Stress and Water

**FIGURE 3:**
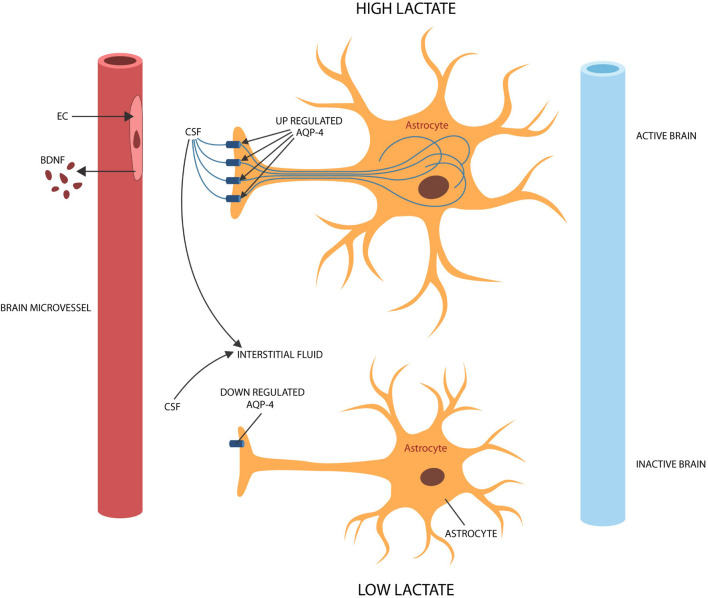
During brain activation and information processing, ISF enters active astrocytes via AQP-4 channels, leaving less fluid in the extracellular space and upregulating lactate (shuttled to neurons). During idle time or slow wave sleep, ISF exits the astrocyte, widening the extracellular space and lowering lactate to facilitate waste clearance. To accomplish their physiological functions, astrocytes require activation by BDNF. Senescent cerebral ECs produce less BDNF, probably leading to astrocyte deactivation and dysfunctional glymphatic clearance.

Cerebral ECs form the BBB with astrocytic end-feet, structures rich in aquaporin-4 (AQP-4) channels that also engender the glymphatic system, a waste disposal apparatus, located between astrocytic processes and ECs (Daneman and Prat, 2015; Krikov et al., 2008). Interstitial fluid (ISF) circulation through this space facilitates the clearance of molecular debris and contributes to the brain-wide diffusion of peptides, neurotransmitters, BDNF and viral particles (Jessen et al., 2015; Plog and Nedergaard, 2018; Figure 3). A growing body of evidence connected that AQP-4 to BDNF and neuroplasticity in the hippocampus, amygdala and mPFC (Szu and Binder, 2016; Huber et al., 2018). On the other hand, dysfunctional AQP-4 channels were associated with depression, insomnia, dysfunctional synaptic plasticity, and impaired fear extinction (Scharfman and Binder, 2013; Berntsen and Rubin, 2014). For example, AQP-4 knockout mice displayed both corticosterone-induced depression and defective memory, symptoms reversed by mifepristone and fluoxetine, linking the brain water circulation to SRDs (Kong et al., 2014; Di Benedetto et al., 2016). Moreover, astrocytic end-feet were demonstrated to contain abundant angiotensinogen, a molecule regulated by AQP-4, connecting this protein to RAS (Wei et al., 2019). Interestingly, ARBs and ACEi were demonstrated to upregulate peritoneal AQP-4, suggesting that these agents may have a similar effect in astrocytic end-feet (Matsushita et al., 2010). In addition, as COVID-19 was reported to target hippocampal astrocytes, the virus likely alters AQP-4 (via ANG II), disrupting synaptic plasticity and fear extinction (Imai et al., 2001; Tavčar et al., 2021).

In our previous work on delirium, we hypothesized that dysfunctional AQP-4, led to impaired information processing, connecting these proteins to neuroplasticity and memory formation (Sfera et al., 2015; Ritchie et al., 2020). This is now supported by data, demonstrating that AQP-4 receptors are essential for brain activation during mental work and deactivation during downtime or sleep, revealing an inverse relationship between memory formation and ISF circulation (Sfera and Osorio, 2014; DiNuzzo and Nedergaard, 2017). Indeed, astrocytic end feet, express the most AQP-4 channels in the entire CNS, playing a key role in brain activation and deactivation (Mader and Brimberg, 2019).

Astrocytes have previously been implicated in PTSD as they regulate synaptic transmission, plasticity, and the formation of aversive memories (Nagelhus and Ottersen, 2013; Saur et al., 2016). To participate in neuroplasticity as well as in the glymphatic circulation, astrocytes require activation by BDNF (Brigadski and Leßmann, 2020). In this regard, PTSD-associated hypermnesia and dissociative amnesia may be traced to astrocytic deactivation (Kol et al., 2020). For example, preclinical studies have associated fear with dysfunctional glymphatic circulation, further connecting the astrocyte to stressful experiences (Li et al., 2020). As astrocytic activation restores the glymphatic circulation, lowering anxiety and fear in animal models, stimulation of these cells may emerge as a therapeutic strategy for SRDs (Martin-Fernandez et al., 2017; Verkhratsky and Nedergaard, 2018; Figure 3). Other preclinical studies have reported that ANG II inhibits GABAergic transmission in the CNS, triggering anxiety and fear, further connecting dysfunctional RAS to SRDs (Li and Pan, 2005; Wang et al., 2018; Zhou et al., 2019). This is significant as it may explain the high prevalence of PTSD in COVID-19 survivors.

Astrocytes have been reported to drive LTP as they shuttle lactate to active neurons, increasing plasticity and memory formation (Hagiwara and Kubo, 2007). Indeed, preclinical studies have associated amnesia with impaired astrocytic lactate transporters, while studies in humans linked psychological stress to the upregulated plasma lactate (Suzuki et al., 2011; Descalzi et al., 2019). Moreover, patients with neuromyelitis optica, a rare autoimmune disease marked by autoantibodies against AQP-4, demonstrated elevated lactate concentrations, suggesting an inverse relationship between glycolysis and the expression of water channels in astrocytic end-feet (Kubera et al., 2012). Along these lines, novel studies have shown that lactate circulates through AQP-4 and that glycolysis is inversely corelated with the expression of water channels (Jarius et al., 2014). Indeed, lactate is upregulated during brain activation, likely to facilitate water entry into astrocytes, while during idle time or slow wave sleep, AQP-4 channels are downregulated to facilitate the glymphatic clearance (Jarius et al., 2014).

Several viruses, including HIV and SARS-CoV-2, were associated with AQP-4 autoantibodies, suggesting that disabling the water channels to upregulate lactate may be a strategy adopted by select pathogens (Lundgaard et al., 2017; Rahimy et al., 2017; Mariajoseph-Antony et al., 2020; Tice et al., 2020; Erickson et al., 2021). Indeed, AQP-4 autoantibodies were found in a subset of COVID-19 patients with neurological symptoms, linking defective water channels to COVID-19 critical illness (Corrêa et al., 2021). In contrast, normal aging was associated with upregulated AQP-4 and loss of glycolysis, further emphasizing the inverse relationship between water channels and brain lactate (Owasil et al., 2020; Corrêa et al., 2021).

Taken together, dysfunctional AQP-4 interferes with RAS and the brain metabolism. As viruses thrive in lactate-rich environments, they may have developed the capability to disable AQP-4 channels, promoting glycolysis and excessive lactate that increases SRD vulnerability.

## Mitochondria and PTSD

As the final part of the manuscript, we are looking into the higher cellular structures, i.e., organelles and in particular mitochondria, their relation to COVID-19, and potential insights into PTSD.

**FIGURE 4:**
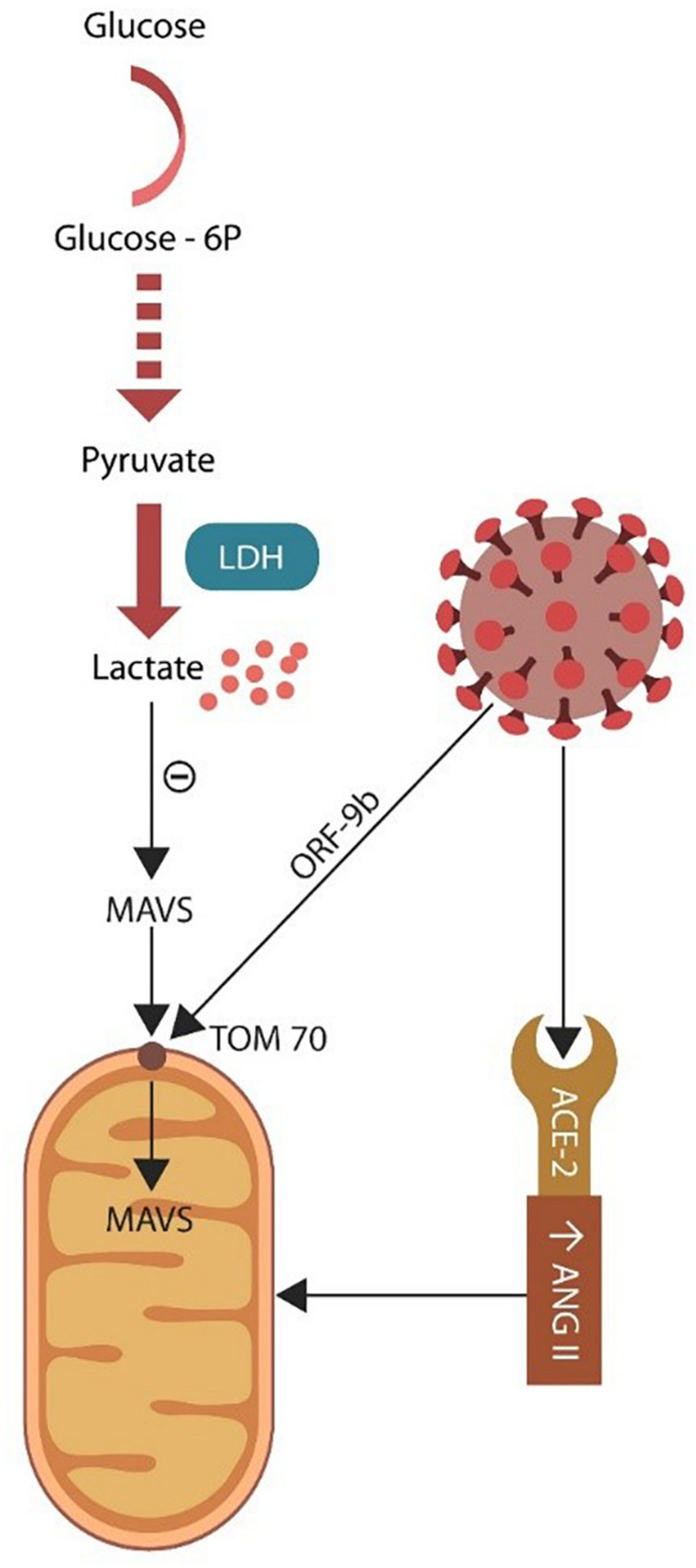
Glycolysis takes place in the cytoplasm where glucose is converted to pyruvate. Under normal circumstances pyruvate enters the mitochondrion and generates ATP via OXPHOS. Damaged mitochondria may be incapable of sustaining OXPHOS, forcing the cell to rely on lactate (generated via LDH). Excess lactate blocks the antiviral MAVS entry into the mitochondrion through the TOM70 pore. SARS-CoV-2 virus targets TOM70 (via ORF-9b), emphasizing the importance of this channel for both antiviral defenses and cellular metabolism.

Mitochondria are dynamic intracellular organelles that participate in multiple physiological functions, including metabolism, ROS generation, cellular senescence, and innate antiviral immunity (Goyal et al., 2017). The latter is initiated by mitochondrial import of antiviral signaling protein (MAVS) through the pore TOM70 (translocase of the outer mitochondrial membrane 70) (Seth et al., 2005; Tang et al., 2014; Gordon et al., 2020). Indeed, most of the mitochondrial proteome is encoded by the nuclear genome and imported into the organelle as precursor proteins. Recently, TOM70 was associated with mitochondrial bioenergetics, indicating that viral exploitation of this protein could also alter cellular metabolism (Liu et al., 2010; Figure 4).

Mitochondria are rich in iron as they house the iron-sulfur clusters (ISCs) and heme prosthetic groups. Since infectious agents, including the SARS-CoV-2 virus may require iron for replication, they target the mitochondrion. However, the host immune system is also dependent on iron for clonal expansion and sequestrates this biometal, initiating the “battle for iron” (Filadi et al., 2018). To extract iron, the SARS-CoV-2 virus may damage the mitochondrion directly via ORF9b and indirectly via bromodomains and ANG II (Gordon et al., 2020; Figure 1). As mitochondria express AT-1R and ET-1Rs, the virus may also disrupt the organelle via ANG II accumulation (Carver, 2018; Ganji and Reddy, 2021). For example, ANG II-upregulated ET-1 damages the organelle by interfering with mitochondrial genome and the cellular metabolism (Dikalov and Nazarewicz, 2013). Indeed, several studies found that ROS upregulate mitochondrial AT-1Rs, while ARBs or ACEi lower ROS, restoring mitochondrial function (de Cavanagh et al., 2003; Chaphalkar et al., 2020).

Psychological stress was shown to upregulate ROS, damaging the mitochondrion that in turn triggers cellular senescence in many cell types, including the cerebral ECs (Dai et al., 2011; Toda and Nakanishi-Toda, 2011; Salim, 2014; Kershaw et al., 2017). On the other hand, preclinical studies have reported that mitochondrial antioxidants, such as elamipretide or SS-31, restore organelles’ homeostasis, restoring endothelial function (Tarantini et al., 2018; Chiao et al., 2020). Along these lines, several studies on combat veterans with PTSD found a direct relationship between symptom severity and the degree of mitochondrial damage, emphasizing the role of these organelles in SRDs (Flaquer et al., 2015; Bersani et al., 2016; Chen et al., 2017d). Moreover, psychological stress-upregulated ROS trigger telomere attrition, a phenomenon encountered in both PTSD and severe COVID-19, linking these conditions to premature aging (Atanackovic et al., 2002; Yegorov et al., 2020). Interestingly, it was reported that aside from AT-1Rs and ET-1Rs, mitochondria also expressed gamma-aminobutyric acid (GABA), glucocorticoid, and monoamine oxidase A and B receptors, further connecting the organelle to SRDs (Krueger, 1995; Mongelli et al., 2021).

### Mitochondria and Lactate

Most cells throughout the body obtain ATP from mitochondria-associated OXPHOS but under hypoxic conditions switch to glycolysis, deriving energy from lactate (Naoi et al., 2006; Owasil et al., 2020). The SARS-CoV-2 virus may target mitochondria to acquire iron and block the import of MAVS, while at the same time it rewires the cellular metabolism to aerobic glycolysis in a Warburg-like effect encountered in malignant cells (Wilson, 2017; Figure 4). Damaged organelles release mitochondrial DNA (mDNA), including copy number (mtDNAcn) and cell-free mitochondrial DNA (cf-mtDNA), emphasizing potential biomarkers for both PTSD and COVID-19 critical illness (Bersani et al., 2016; Chauhan et al., 2019; Scozzi et al., 2021; Trumpff et al., 2021).

As the SARS-CoV-2 virus thrives on lactate, it preferentially targets ECs, that under normal circumstances, derive most of their energy from glycolysis. In addition, as the virus induces EC senescence, it may upregulates lactate further, generating a friendly microenvironment for its replication. Excessive brain lactate, as discussed above, likely triggers PTSD symptoms by activating ASIC1a and downregulating AQP-4 (Smoller et al., 2014; Quagliato et al., 2018; Li et al., 2020).

Propranolol, an established glycolysis inhibitor, has been utilized in PTSD for decades as it facilitates the extinction of fear learning, linking this cognitive defect to excessive lactate (Vaiva et al., 2003; Brohée et al., 2018; Ganji and Reddy, 2021). In addition, propranolol protects ECs by restoring mitochondrial integrity, increasing PTSD resilience (Giustino et al., 2016). Propranolol also blocks the Warburg effect, disrupting the energy supply of both malignant and virus-infected cells, emphasizing the anticancer and antiviral properties of this drug (Iwai et al., 2002; Lucido et al., 2018; Barbieri et al., 2020; Vasanthakumar, 2020).

Aside from its role as a metabolite, lactate is also a signaling molecule that interacts with G-protein-coupled receptor 81 (GPR81), increasing angiogenesis and opposing ECs senescence (Morland et al., 2015, 2017). Interestingly, preclinical studies reported that GPR81 agonists trigger anxiety via cAMP, suggesting that blocking these receptors could be therapeutic in SRDs (Morland et al., 2015; Shan et al., 2020). Interestingly, a cAMP transcription factor, cAMP response element-binding (CREB) protein, was implicated in PTSD, and can be inhibited by dopamine blockers (Tsang and Lal, 1977; Martini et al., 2013; Keil et al., 2016).

Another mitochondria-protective agent with therapeutic potential in PTSD is dichloroacetate (DCA), a lactate inhibitor with established anticancer and antiviral properties (Kho et al., 2019). Despite these benefits, DCA use in PTSD is likely limited by its serious adverse effects such as peripheral neuropathy and delirium (Table 2).

Mitochondrial transplant, used with some success in pediatric patients with Pearson’s syndrome, may also benefit PTSD patients by replacing damaged organelles (Emani and McCully, 2018). However, as of this time, it is unclear whether transplanted mitochondria can survive in the extracellular environment or selectively enter in the defective cells, indicating that this intervention is not ready for clinical practice at this time. However, another procedure based on introducing functional mitochondria directly into dysfunctional cells may be more promising for PTSD (Gomzikova et al., 2021).

### Mitochondria and Neurosteroids

Neurosteroids, synthesized in the CNS, adrenals, and gonads, are agonists at GABA-A receptors, that play a major role in regulating multiple brain signaling pathways (Lightowlers et al., 2020). Mitochondria initiate steroidogenesis by importing cholesterol to synthesize pregnenolone, a precursor molecule, that exits the organelle and is converted to progesterone, pregnanolone, and allopregnanolone in the cytoplasm (Zorumski et al., 2013).

Women with PTSD demonstrated low CSF levels of allopregnanolone and pregnanolone, further linking this disorder to mitochondrial dysfunction (Rasmusson et al., 2006; Almeida et al., 2021). Interestingly, allopregnanolone, also known as brexanolone, was approved by the Food and Drug Administration (FDA) for the treatment of postpartum depression (Pineles et al., 2018; Morrison et al., 2019). Earlier studies found that allopregnanolone improved the BBB permeability, indicating potential benefits for senescent endothelia and PTSD (brexanolone is currently in clinical trials for this disorder) (NCT04468360). Moreover, allopregnanolone was found therapeutic in PTSD and severe COVID-19, as it might reverse some aspects of endothelial senescence (Lüscher and Möhler, 2019). In addition, progesterone was reported beneficial for both COVID-19 and PTSD, possibly by increasing NO in cerebral ECs (Balan et al., 2019; Ney et al., 2019; Seligowski et al., 2020). Indeed, as mentioned above, NO pathology contributes to PTSD and is currently in clinical trials for COVID-19 (Ghandehari et al., 2021) (NCT04601077) (Table 2).

Taken together, neurosteroids may be therapeutic for PTSD and COVID-19 as they improve mitochondrial function and restore endothelial integrity.

### Mitochondria and Bromodomains

The E (envelope) antigen of SARS-CoV-2 virus hijacks BRD2 and BRD4, possibly damaging mitochondria that in return trigger cellular senescence, glycolytic states, and impaired antiviral immunity (Gordon et al., 2020). In animal models, defective BRD4 was associated with impaired synaptic plasticity and memory formation, likely linking this protein to PTSD-related amnesia, depression, and anxiety, while at the same time indicating that BRD4 inhibitors may be therapeutic for SRD (Bruenig et al., 2017; Wang et al., 2021). The SARS-CoV-2 virus may target BDR4 as it regulates antiviral immunity, including the interferon-gamma (IFN-gamma), a molecule previously associated with both PTSD and affective disorders (Bruenig et al., 2018; Gibbons et al., 2019; Huang et al., 2021). Interestingly, in humans, IFN-gamma was shown to drive EC senescence, linking COVID-19 further to premature cellular aging (Bam et al., 2016).

Bromodomains are epigenetic readers that regulate the chromatin landscape in cell nucleus, influencing multiple genes. For example, BRD4 regulates the expression of mitochondrial proteins encoded in nuclear DNA, including the ones in charge of metabolism, suggesting that viral exploitation of this protein may inhibit OXPHOS and activate glycolysis (Kim et al., 2009, 2020). Indeed, BRD4 engenders the glycolytic states associated with senescent cells and their secretome, the senescence-associated secretory phenotype (SASP) (Barrow et al., 2016). On the other hand, BRD4 inhibitors, including JQ1 or SF2523, act as senolytics by promoting the clearance of senescent and virus-infected cells (Tasdemir et al., 2016; Go et al., 2021; Table 2). Moreover, as senolytics were found therapeutic against idiopathic pulmonary fibrosis, a BRD4-associated condition, these agents may be helpful to SRD patients (Tang et al., 2013; Acharya et al., 2021). As senolytic agents facilitate the clearance of all damaged cells, including the malignant and virus-infected ones, they are currently being evaluated for COVID-19 (Hu et al., 2020; Go et al., 2021).

Taken together, this data links SRDs to dysfunctional mitochondria and EC senescence, while, on the other hand, BRD4 inhibitors protect the mitochondrion, preempting premature endothelial aging.

### Molecular Mimicry and Inflammation

Recent bioinformatic studies established that some SARS-CoV-2 antigens mimic human proteins expressed by neurons, astrocytes, and CECs, predisposing to PTSD. For example, viral N protein shares amino acid residues with the human zonula occludens-1 (ZO-1) and solute carrier family 12 member 6 (SLC12A6), molecules linked to BBB permeability (Hu et al., 2020). In addition, tryptophan 5-hydroxylase 2 (TPH2), an enzyme involved in serotonin synthesis, was found to have a strong mimicry with the S protein of SARS-CoV-2, potentially contributing to anxiety and depression (Yapici-Eser et al., 2021). This is significant as TPH2 plays a major role in PTSD, as well as in premature ECs senescence (Goçi Uka et al., 2019; Wu, 2021). Moreover, the molecular mimicry between the SARS-CoV-2 protein S and human anti-inflammatory proteins was associated with inflammation, a pathology demonstrated in PTSD (Kanduc, 2020). As mitochondrial damage can directly activate inflammasomes, leading to inflammation, the virus may utilize several mechanisms to ignite this pathology (Zhou et al., 2011).

## Limitations/Gaps/Challenges

Epidemiologic studies have associated some viruses with anxiety, depression, and PTSD however, only a handful of researchers have systematically studied this connection. More studies are needed to elucidate the interaction between communicable diseases and psychiatric disorders, including SRDs. To fill the knowledge gap between endothelia and PTSD, a combination of different expertise domains is required, an endeavor often difficult to accomplish in clinical context.

Despite these drawbacks, available studies appear to support the concept that, like psychosocial trauma, some viruses promote cellular senescence in many cell types, including ECs and T cells, emphasizing the role of premature aging in these conditions (Justice et al., 2019; Saleh et al., 2020). Indeed, trauma-focused psychotherapy, was shown to restore the physiological T cell phenotypes and reduce PTSD symptoms, suggesting that psychotherapy may have a place in infectious diseases, including the long COVID-19 (Prather et al., 2018). When this is considered together with the fact that IFN-gamma, a regulator of T cell aging, is depleted in both PTSD and COVID-19, a bromodomain-mediated epigenetic mechanism is emerging (Morath et al., 2014; De Biasi et al., 2020). Indeed, several studies have pointed to the fact that in old brains’ T cells express abundant IFN-gamma, a molecule that promotes BBB leakage (Tewari et al., 2007; Michopoulos et al., 2017; Bonney et al., 2019; Dulken et al., 2019). Although some studies failed to demonstrate endothelial dysfunction in PTSD, they connected this disorder to impaired coagulation, a pathology validated by several other studies (von Känel et al., 2008; Robicsek et al., 2011). Systematic studies in this area would be beneficial, especially in the age that has seen increased incidence of viral infections and SRDs.

## Conclusion

COVID-19 has been associated with a high PTSD prevalence, suggesting that aside from the psychological burden of the disease itself, the virus may directly interfere with the stress-processing brain areas. The virus-host interactome reveals several pathogen-induced states, such as cellular senescence and associated glycolysis, that facilitate viral replication, while at the same time disrupt the BBB. The virus likely increases PTSD susceptibility as, even in the absence of viral infection, this disorder has been associated with EC senescence and glycolytic states. This two-strike model not only may explain the high comorbidity of COVID-19 and PTSD, but also opens vistas for novel interventions, including ARBs, ACEi, ASIC1a blockers, senolytics, furin inhibitors, BRD4 inhibitors and anti-glycolytic interventions.

## Author Contributions

All authors listed have made a substantial, direct and intellectual contribution to the work, and approved it for publication.

## Author Disclaimer

Where authors are identified as personnel of the International Agency for Research on Cancer/WHO, the authors alone are responsible for the views expressed in this article and they do not necessarily represent the decisions, policy or views of the International Agency for Research on Cancer/WHO.

## Conflict of Interest

The authors declare that the research was conducted in the absence of any commercial or financial relationships that could be construed as a potential conflict of interest.

## Publisher’s Note

All claims expressed in this article are solely those of the authors and do not necessarily represent those of their affiliated organizations, or those of the publisher, the editors and the reviewers. Any product that may be evaluated in this article, or claim that may be made by its manufacturer, is not guaranteed or endorsed by the publisher.

## Abbreviations

ANGII: angiotensin II
ET-1: endothelin-1
PAI-1: plasminogen activator inhibitor 1
RAS: renin-angiotensin system
Sig-1R: Sigma-1 receptor
FCS: furin cleaving site
CECs: cerebral endothelial cells
ECs: endothelial cells
BDNF: brain derived neurotrophic factor
MAVS: mitochondrial antiviral signaling
PTSD: posttraumatic stress disorder
SRDs: stress related disorders
HPA: hypothalamic-pituitary axis
ARBs: angiotensin receptor blockers
ACEi: angiotensin converting enzyme inhibitors
ACE-2: angiotensin converting enzyme 2
OXPHOS: oxidative phosphorylation
BRDs: bromodomains
LTP: long term potentiation
LTD: long term depression
ASC1a: acid-sensitive ion channel-1a
TrkB: tropomyosin receptor kinase B
AQP-4: aquaporin-4.
